# Vection induced by a pair of patches of synchronized visual motion stimuli covering total field of views as small as 10 square-degrees

**DOI:** 10.1177/20416695231201463

**Published:** 2023-09-24

**Authors:** Coskun Joe Dizmen, Richard H.Y. So

**Affiliations:** 125731 American University of the Middle East, Kuwait; Hong Kong University of Science and Technology, Hong Kong

**Keywords:** vection, visual perception of locomotion, small field of view

## Abstract

Vection (illusion of self-motion) is known to be induced by watching large field-of-view (FOV) moving scenes. In our study, we investigated vection induced by small FOV stimuli. Three experiments were conducted in 45 sessions to analyze vection provoked by moving scenes covering total FOVs as small as 10 square-degrees. Results indicated that 88% of the participants reported vection while watching two small patches of moving dots (1° horizontal by 5° vertical, each) placed on the left and right sides of the observers. This is less than a quarter of the total visual area of two Apple Watches viewed at a distance of 40 cm. Occlusion of the visual field between the two display patches significantly increased the levels of rated vection. Similarly, increasing the speed of the moving dots of the two display patches from about 5 to 25 °/sec increased the levels of rated vection significantly. The location of the two patches in the horizontal visual field did not affect the vection perception significantly. When the two straight stripes of dots were moving in opposite directions, participants perceived circular vection. The observers connected the two stimuli in their minds and perceived them as parts of a single occluded background. The findings of this study are relevant to the design of mobile devices (e.g., smartphones) and wearable technology (e.g., smart watches) with small display areas.

## Foundation

Vection is a stationary observer's illusion of self-motion. Researching vection is not only important for understanding human perception, but also for improving the quality of human computer interactions. On the one hand, vection perception can increase immersion and sense of presence in a virtual environment (Lin et al., 2002). On the other hand, prolonged vection can elicit undesirable symptoms ranging from slight-uneasiness to nausea and vomiting, that is, visually induced motion sickness (Kennedy et al., 2010; Laessoe et al., 2023). Understanding the mechanisms of vection will enable designers to control their effects: using vection appropriately in, for example, video games, while avoiding unwelcome side effects in other contexts. Therefore, the underlying mechanisms of vection have been investigated by many researchers (So & Ujike, 2010).

A review of literature indicates that vection studies have utilized different forms of visual stimulation including moving physical objects like optokinetic drums (e.g., Brandt et al., 1973), stimuli displayed on light-emitting monitors (e.g., D’Amour et al., 2021; Johansson, 1977), projected optic flows (e.g., Trutoiu et al., 2009), and head-mounted displays (e.g., Palmisano et al., 2017; So et al., 2001). As the stimuli differed across studies, the field-of-view (FOV) of the stimuli differed as well. Since the perception of visual motion mainly relies on the magnocellular visual pathway, which is mostly connected to the peripheral visual field, it is not surprising that most of the past studies on vection used stimuli covering large FOVs (Solari et al., 2019). The smallest area used to stimulate each eye of the participant in the literature is 1° by 47°, approximately (i.e., two stripes of 47 square-degrees) (Johansson, 1977). Indeed, the minimum condition for vection induction has been a subject of interest. Besides the minimum FOV, Nakamura (2013) examined the minimum number of movie frames that can induce vection. The focus of this study is the smallest FOV for vection induction.

In today's world, much information is obtained from small screens like mobile phones and smart watches. To the best of our knowledge, there is no published scientific experiment that tests whether vection can be invoked while watching screens as small as smart watches. Our experiments will also attempt to fill in this gap.

## Motives of Our Study

Johansson (1977) reported that more than half of his subjects experienced vection when exposed to two stripes of moving dots of FOV of only 47 square-degrees. In his study, subjects were exposed to moving dots of larger FOVs first, and then the stimuli FOV was progressively reduced to 47 square-degrees on each side. Considering how small the stimulation area was and that no other researchers, except for us in a preliminary discussion in VIMS 2015 (Dizmen & So, 2015), have reported vection at such small FOVs since 1977, one may argue that Johansson's approach of inducing vection with large stimuli and then reducing the stimuli size gradually might have encouraged subjects to over-report vection. The first objective of our study is to examine whether experiencing vection with larger stimuli is a necessary precondition for experiencing vection with Johansson's (1977) small stimuli.

The other objectives of our study were to examine the effects of (i) frontal occlusion, (ii) stimuli speed, (iii) vection type (linear vs. circular), (iv) vertical FOV of the stimuli, and (v) stimuli location on vection. The motives for choosing these specific factors are discussed briefly below.

### Frontal Occlusion

Johansson's stimulation was an elevator simulator. The subjects judged whether they felt no self-motion, or vertical self-motion (either upward or downward). Johansson used movable screening plates to adjust the stimuli area and cause “the window effect.” While Johansson's (1977) subjects had an open view of their laboratory room, only 7% of the subjects reported vection at 47 square-degrees stimuli. The percentage of subjects who reported vection at 47 square-degrees when they occluded their frontal vision with their hands, however, was 80%.

It is believed that the display a person perceives in the background controls whether the person experiences vection (Ohmi et al., 1987). Having a stationary object in the foreground while the background has a moving pattern causes vection more easily compared to having no stationary objects (Howard & Howard, 1994). When foreground stimulation is moving slowly in the same direction as the background or quickly against it, the vection is inhibited (Nakamura & Shimojo, 1999). Nakamura (2006) found that increasing the size of the static foreground stimulation increases the level of vection facilitation brought by the static foreground while viewing the moving background. Considering these findings, one may expect that viewing a room which has many static objects—such as furniture—would cause higher levels of vection compared to blocking the frontal vision with hands. However, Johansson's (1977) results show just the opposite of that expectation. The explanation may be that unobstructed frontal vision led the room to be perceived as background and the stimuli as foreground; whereas occluding the frontal vision reduced the visual cues in the room and led observers to perceive the moving patches of stimuli as parts of stationary walls of an elevator shaft, that is, a background.

In Johansson's study (1977), the ceiling light was on and the experiment room had many visual cues in the form of furniture and equipment. In combination with frontal occlusion, the visual cues can be reduced further by turning off the ceiling light which in turn leads to more compelling vection (see Dizmen & So, 2015). Therefore, in our experiments we displayed the stimuli on monitors and used screening plates to induce “the window” effect, and we kept the ceiling lights off. By incorporating frontal occlusion as an independent factor of our Experiment 1, we aimed to investigate the effect of frontal occlusion while the ceiling lights were off, by asking our subjects to hold a cardboard—rather than asking them to block their frontal vision with their hands only.

### Stimuli Speed

The stimuli speed used in Johansson's study (1977) was 10.5°/sec. He wrote, “Within certain limits the speed of motion was not critical for the effect studied.” However, past studies such as Berthoz et al. (1975), Andersen and Braunstein (1985), and Howard and Howard (1994) suggest that the stimuli speed may affect the perception of self-motion. Our preliminary trials with these stimuli also showed that speed might affect vection. Therefore, we used different speeds in our study.

### Vection Type (Linear vs. Circular)

The stimuli used in our Exp1 were two narrow stripes (1° in width and 47° in height) of moving dots adopted from Johansson (1977). Similar to Johansson's study, the dots in our Exp1 were moving in the same direction, inducing linear vection. In Johansson's own words “elevator motion” was simulated with the two stripes moving in the same direction. In our experiments, some participants described their feeling of self-motion like “moving in space” vertically rather than thinking of a motion which is restricted in an elevator shaft. One reason for this might be the fact that our experiments were conducted in a relatively darker room compared to Johansson's. When the two stimuli patches were going in the same direction, our subjects consistently experienced linear vection (either upward or downward depending on the direction of the stimuli).

Our initial exploratory tests showed that when the dots on one stimulation patch were going up and the ones on the other patch were going down, some subjects had a sensation of circular vection—namely roll vection. In Exp2, we tested whether roll vection caused by two patches of dots moving in opposite directions is stronger than linear vection caused by two patches of dots moving in the same direction.

### Vertical FOV of the Stimuli

The stimuli used in Johansson's experiment were 1° in width and 47° in height, which is not a common shape or size for displays. As our study intended to investigate minimal FOV conditions that induce vection, we also checked the effect of reducing the vertical FOV while keeping the horizontal FOV 1°. In this way, we would be able to see whether a screen size comparable to smartphones or smart watches/bands might be able to induce a similar vection.

### Stimuli Location

Brandt et al. (1973) hypothesized that peripheral vision plays a more important role in vection perception than central vision. However, Post (1988) found no significant difference between central and peripheral stimulation using a circular vection-provoking stimulation. Nakamura and Shimojo (1998) used a linear vection-provoking stimulation and found similar results as Post's (1988). Whether peripheral vision is dominant or not may be affected by a range of factors such as spatial frequency of the stimuli (Palmisano & Gillam, 1998), and whether there is a point of fixation or not (Tarita-Nistor et al., 2006). Although the majority of past studies suggest that the location of the stimuli may not be significant, conflicting findings exist. In this study, we examined the influence of location in order to both “cover all bases” and possibly contribute to resolving the controversy.

In Johansson's (1977) study, no participant among 20 reported vection when the central vision was stimulated; however, most participants experienced vection when the stimuli were located in their peripheral visual fields. Our preliminary tests were in accordance with Johansson's findings, supporting the “peripheral dominance” theory as valid for this specific stimulation. However, the question of whether some segments of the peripheral visual field are more crucial to the perception of vection induced by this specific stimulation remained unanswered. In Exp3 (described below), we studied this by testing the effects of placing the vection-provoking stimuli at 30°, 45°, or 70° horizontal locations.

## Methods

### Subjects

The subjects of the experiments were recruited from a pool of 29 self-reported healthy people whose ages ranged from 18 to 31, with a median of 23 years. Experiments 1, 2, and 3 were attended by 13, 16, and 16 subjects, respectively. All subjects were tested to have normal or corrected-to-normal vision according to 20/20 protocol. All experiments were approved by the Committee for Research Practices of the Hong Kong University of Science and Technology.

### Experimental Setup

**Figure 1.**
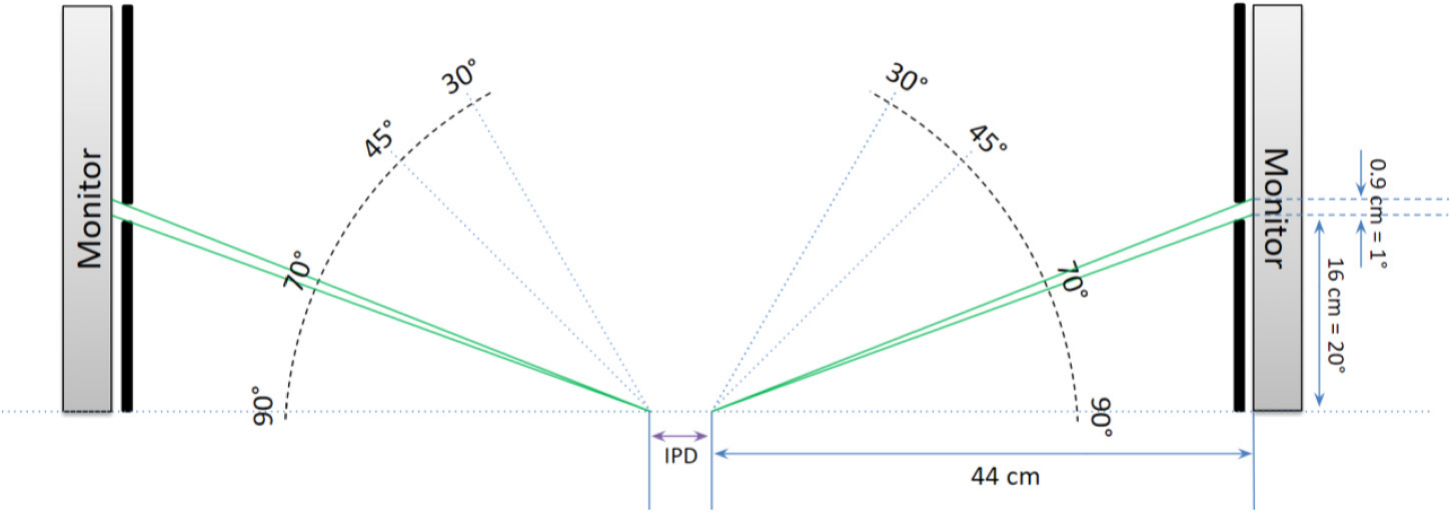
Top view of the experimental setup. In Exp1 and Exp2, only the horizontal location of 70° was used. In Exp3, the horizontal locations of 30°, 45°, and 70° were tested by moving the monitors’ locations. In all experiments, horizontal stimulation FOV was 1° on each side of the subject as shown within the green line segments in the figure. IPD, inter-pupillary distance.

**Figure 2.**
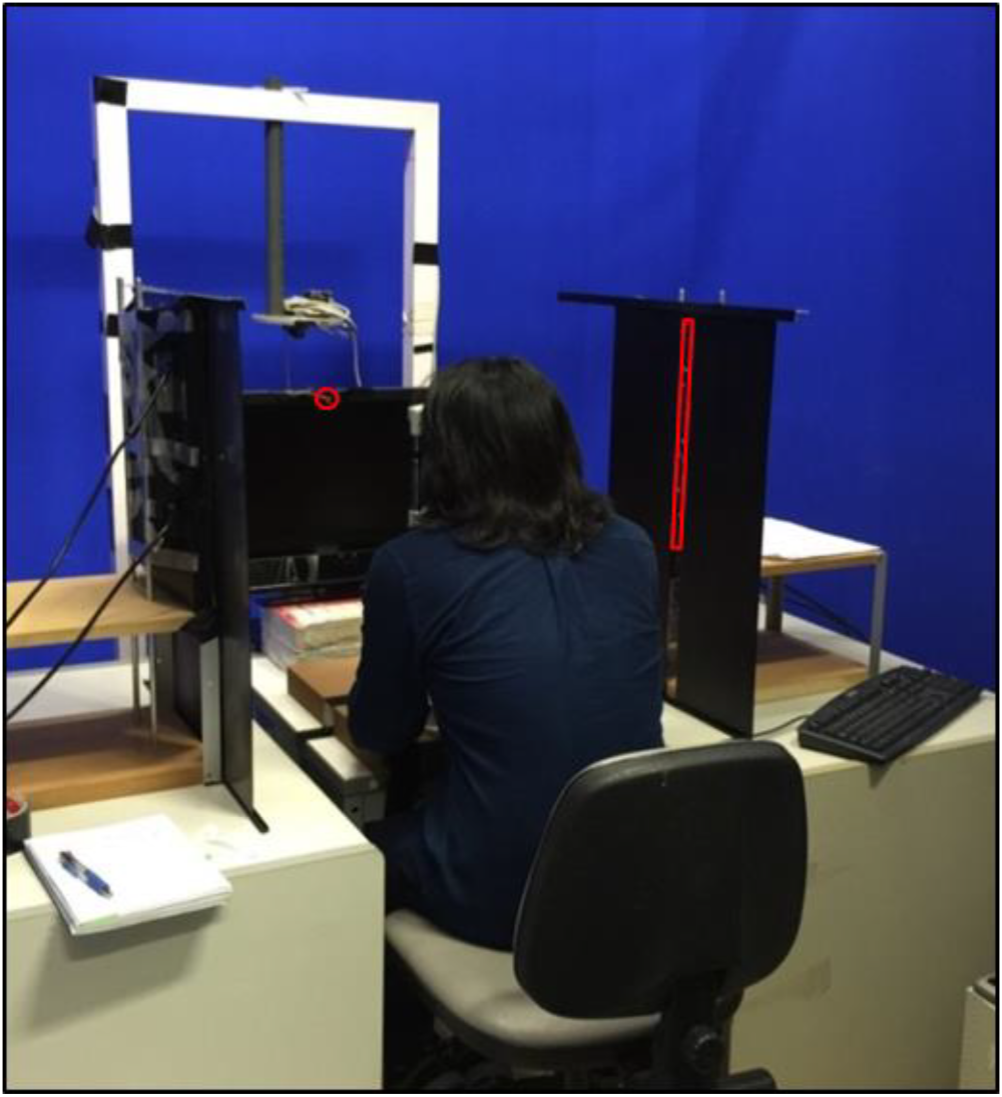
Annotated picture of setup of Experiment 1. The red rectangle on the right and the red circle in front of the subject were added to the picture to indicate the stimulus area and the eye fixation point, respectively.

**Figure 3.**
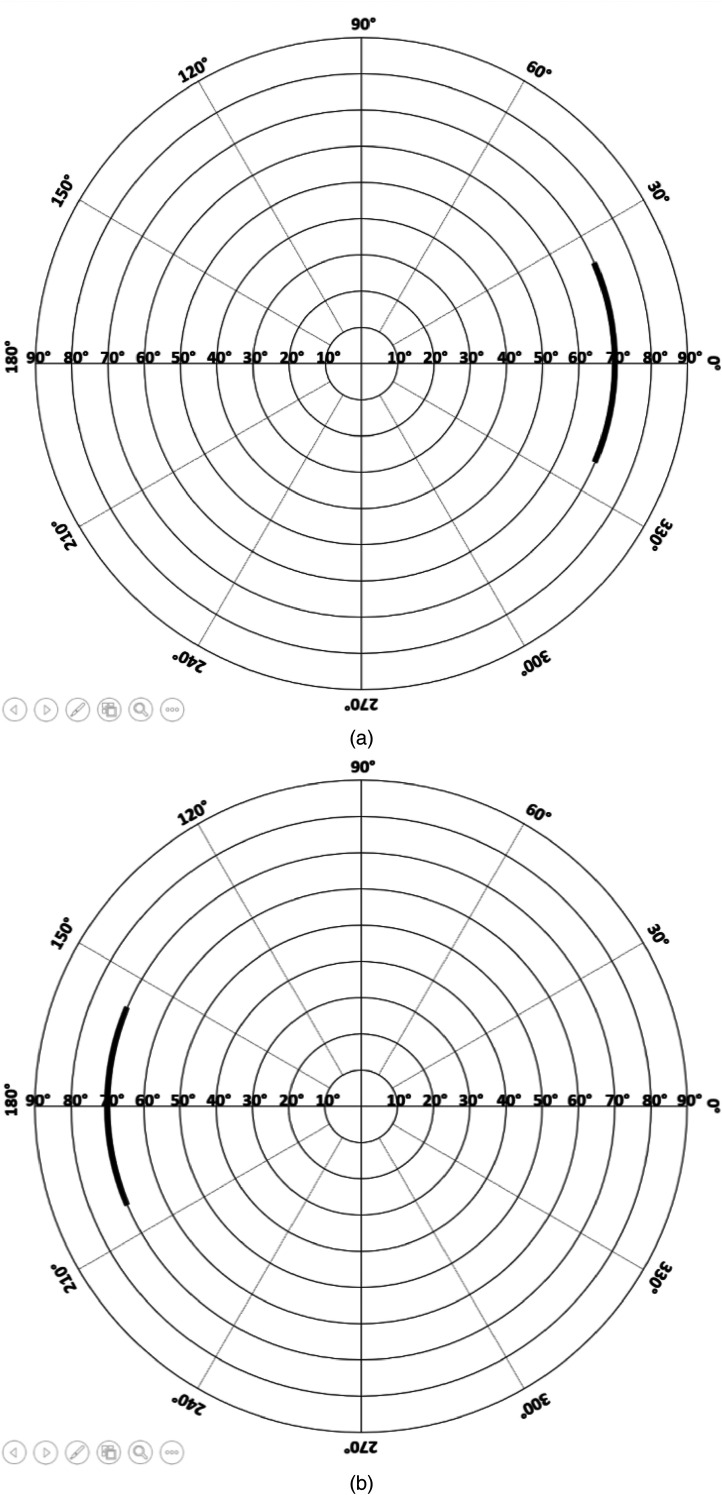
(a) Perimetric chart of left eye in Exp1. The stimulus, which covers 1° in horizontal and 47° in vertical, is on the nasal side. (b) Perimetric chart of right eye in Exp1. The stimulus, which covers 1° in horizontal and 47° in vertical, is on the nasal side.

The stimuli of vertically moving white dots were displayed on a black background with two monitors (Philips 192E1SB/69), one on each side of the subject. The size and speed of all dots were the same during each treatment. Each white dot was rectangular in shape, and approximately 0.5 cm in the horizontal direction and 0.3 cm in the vertical direction. The visible horizontal area of each monitor was set to 0.9 cm, corresponding to a horizontal FOV of 1°. The stimuli on each monitor was 41 cm in height, covering 47° vertical FOV at 70° horizontal location (cf. Johansson, 1977) (see Figure 1). The monitors were parallel to the sagittal plane of the subject's body. The stimuli FOV was kept constant throughout the Experiment 1 (see Figures 2 and 3(a) and (b)).

In half of the trials of Exp1, the participants held a cardboard. In the trials without the cardboard, the subjects were instructed to stare at an LED located 60 cm away at about the same height as their eyes. For the trials in which the subjects held a cardboard, the subjects were only asked to look within the frame of the cardboard, where no eye fixation point existed. The cardboard was black with the dimensions of 35 cm×28.5 cm. Subjects were instructed to hold it with both hands in front of their eyes so that they could see almost nothing but the experimental stimuli with their peripheral vision. In Exp2 and Exp3, the cardboard was fixed in front of the participants.

### Procedures

Subjects were tested individually. All subjects were exposed to all conditions.

The experimental room was dark except for dim light emitted from the stimuli on a pair of monitors. Once inside the room, subjects were allowed a 20-min eye adaption period.

When ready for testing, the subject placed his/her head on a chin-rest for stability. The height of the chin-rest was adjusted so that the center of the stimuli was at the subject's eye level.

Johansson (1977) wrote that the vection induced by his stimuli had an “all-or-none character.” He only counted the subjects who reported the perception of “real elevator motion” rather than “as if sitting in a moving elevator.” Our preliminary tests suggested that the vection perception of our stimuli was not perfectly in “all” or “none” categories. Therefore, we employed a subjective vection rating scale (0 to 6: Allison et al., 1999; see Table 1) to measure the magnitude of the vection. We also recorded the vection onset times (0 to 120 s). If the subject reported vection, the onset time and subjective ratings were reported, and then the stimuli were stopped. If the subject didn’t experience vection within the first 120 s of a trial, a hypothetical vection onset time of 121 s and a subjective rating of 0 were used for data analysis purposes. Between trials, the subject was allowed a rest period regardless of whether vection was experienced or not.

**Table 1.** Subjective vection rating scale (adapted from Allison et al., 1999).

Vection rating scale		
Rating	Definition	Subjective description
0	Only dots are moving	I feel that I am stationary, and only the dots are moving.
1	Dots’ speed >> My speed	I feel that I am moving a bit but dots are moving much more.
2	Dots’ speed > My speed	I feel that I am moving but dots are moving more.
3	Dots’ speed = My speed	I feel that I am moving at the same speed as the dots.
4	Dots’ speed < My speed	I feel that dots are moving but I am moving more.
5	Dots’ speed << My speed	I feel that dots are moving a bit but I am moving much more.
6	Only I am moving	I feel that the dots are stationary, and only I am moving.

The conditions were presented to the subjects in random order. In Exp1, every condition was tested by each subject twice. In Exp2 and Exp3, every condition was tested only once.

### Design of Experiments

The first and foremost motivation of our first experiment was to check whether two stripes of 1° by 47° moving dots can induce vection when the subjects were not conditioned to perceive vection through larger FOVs (such as 10° by 47° like Johansson's experiment) immediately before the trial.

Secondly, in Exp1, we wanted to examine the effects on vection of (i) frontal occlusion (holding a cardboard to block the frontal vision versus staring at an LED with an open view), and (ii) stimuli speed. We tested two speed levels, namely low (4 cm/sec ≈ 5°/sec) and high (21.4 cm/sec ≈ 25°/sec).

After establishing a baseline with Experiment 1, we fixed the frontal occlusionand speed factors and tested other variables in Exp2 & Exp3. Table 2 presents the dependent variables, independent variables, and control factors of each experiment.

**Table 2.** Design of each experiment (FOV refers to the size of the stimuli, whereas location refers to the eccentricity of the stimuli).

	Dependent variables	Independent variables	Control factors
**Exp. 1:**	◦ Subjective vection rating, ◦ Vection onset time.	◦ Stimuli speed: ◾ low (≈5°/sec) ◾ high (≈25°/sec) ◦ Presence of cardboard: ◾ present ◾ not present	◦ Horizontal FOV (=1°), ◦ Horizontal location (70° from the straight gaze), ◦ Vertical FOV (=47°), ◦ Vertical location (center of the stimuli at eye level), ◦ Stimuli direction (both stimuli in the same direction).
**Exp. 2:**	◦ Subjective vection rating, ◦ Vection onset time.	◦ Vertical FOV: ◾ 5° ◾ 10° ◾ 20° ◾ 40° ◦ Stimuli direction: ◾ both stimuli in the same direction ◾ the two stimuli in opposite directions	◦ Stimuli speed (≈25°/sec), ◦ Presence of cardboard (present, fixed in front of the subject), ◦ Horizontal FOV (=1°), ◦ Horizontal location (70° from the straight gaze), ◦ Vertical location (center of the stimuli at eye level).
**Exp. 3:**	◦ Subjective vection rating, ◦ Vection onset time.	◦ Horizontal location: ◾ 30° ◾ 45° ◾ 70° ◦ Vertical FOV: ◾ 5° ◾ 40° ◦ Stimuli direction: ◾ both stimuli in the same direction ◾ versus the two stimuli in opposite directions	◦ Stimuli speed (≈25°/sec), ◦ Presence of cardboard (present, fixed in front of the subject), ◦ Horizontal FOV (=1°), ◦ Vertical location (center of the stimuli aligned with the eye level).

In Experiment 1, the two stimuli were going in the same direction only, causing linear vection. However, in half of the trials of Experiments 2 and 3, the two stripes were going in opposite directions, causing circular vection.

The order in which participants took the trials was randomized. The area of each stripe was constant throughout each trial.

### Statistical Methods

As our data were far from being normally distributed (*p* < .05, Anderson-Darling Test), parametric tests such as ANOVA may give biased results (Boone & Boone, 2012; Lantz, 2013). Therefore, the nonparametric tests Friedman and Wilcoxon Signed-Rank Tests were used in our study.

## Results

### Results of Experiment 1

**Figure 4.**
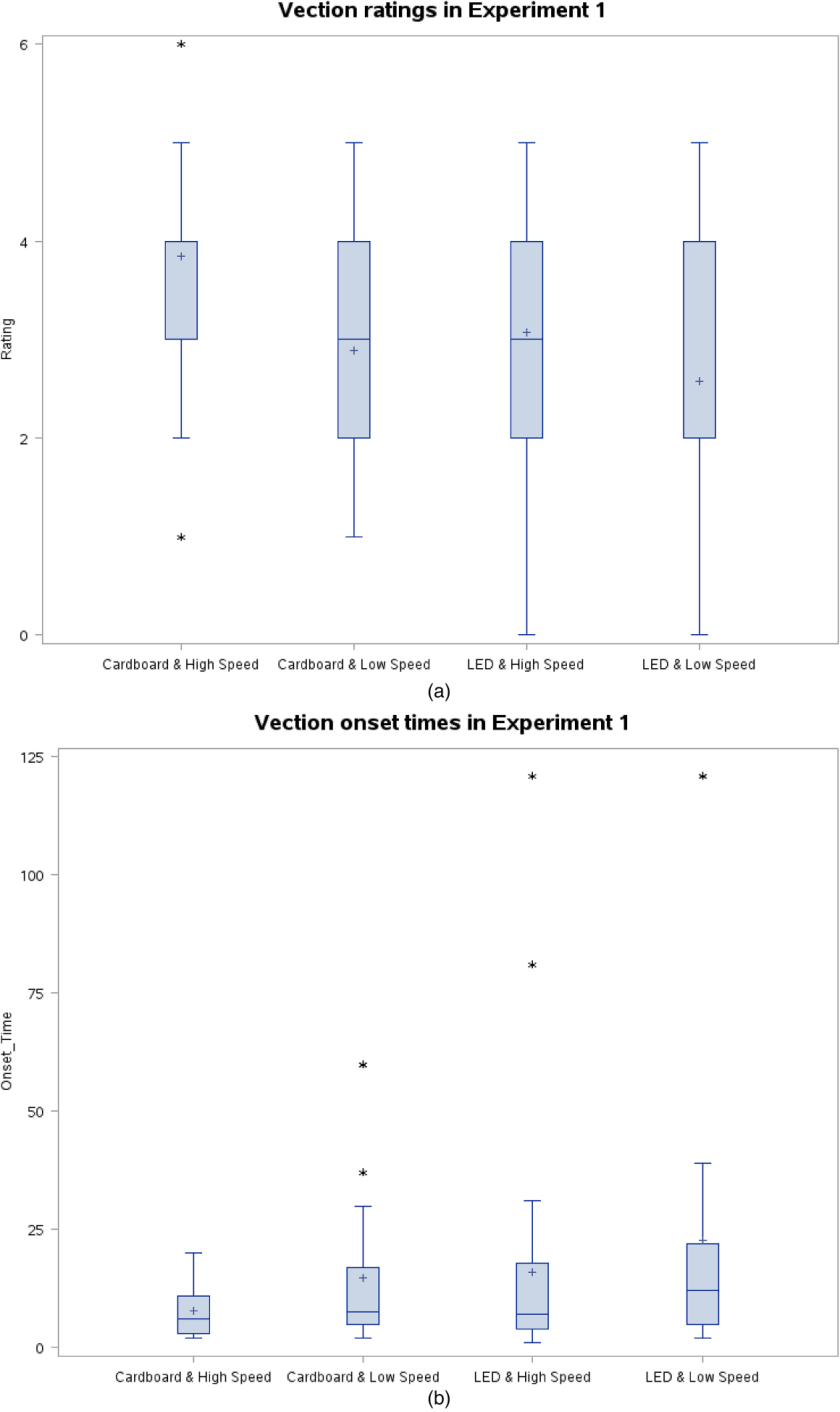
(a) Vection ratings in Exp1. Lower and upper edges of the box correspond to the lower and upper quartiles, respectively. The horizontal line in/on the box and cross represent the median and mean values, respectively. The whiskers extend till the data point within ±1.5 interquartile range. * indicates one or more outliers of the same value. (b) Vection onset times in Exp1 (3 trials did not result in vection. These trials may be assumed to have had vection onset time of infinity and are excluded from the boxplot. However, to keep the graph comprehensive yet simple, we assigned 121 s to these trials because 121 is the smallest whole number that is still bigger than duration of each trial, that is, 120 s). Lower and upper edges of the box correspond to the lower and upper quartiles, respectively. The horizontal line in/on the box and cross represent the median and mean values, respectively. The whiskers extend till the data point within ±1.5 interquartile range. * indicates one or more outliers of the same value.

In 97.1% of the trials, vection was reported. The median vection rating of all trials was 3, and the median vection onset time was 7.5 s. This proves that even after removing the bias from Johansson's study, it is possible to provoke vection with two stimuli stripes, each covering 1° by 47° FOV. Figures 4(a) and (b) depict the results in box plot form.

Holding a cardboard to block the central vision (compared to staring at an LED with open view) caused significantly shorter onset times (*p* < .05; Wilcoxon Signed-Rank Test), and significantly higher vection ratings (*p* < .05; Wilcoxon Signed-Rank Test).

For the higher speed stimuli, the vection onset times were shorter (*p* < .05; Wilcoxon Signed-Rank Test) and the vection ratings were higher (*p* < .05; Wilcoxon Signed-Rank Test).

### Results of Experiment 2

Linear vection had shorter onset times (*p* < .05; Wilcoxon Signed-Rank Test), and higher vection ratings (*p* < .05; Wilcoxon Signed-Rank Test) than the circular vection. It is interesting that all 16 participants experienced circular vection—roll vection—at least once while viewing a pair of straight stripes of dots moving in opposite directions. Subjects reported that they were mentally perceiving the two patches of moving dots as a single pattern.

**Figure 5.**
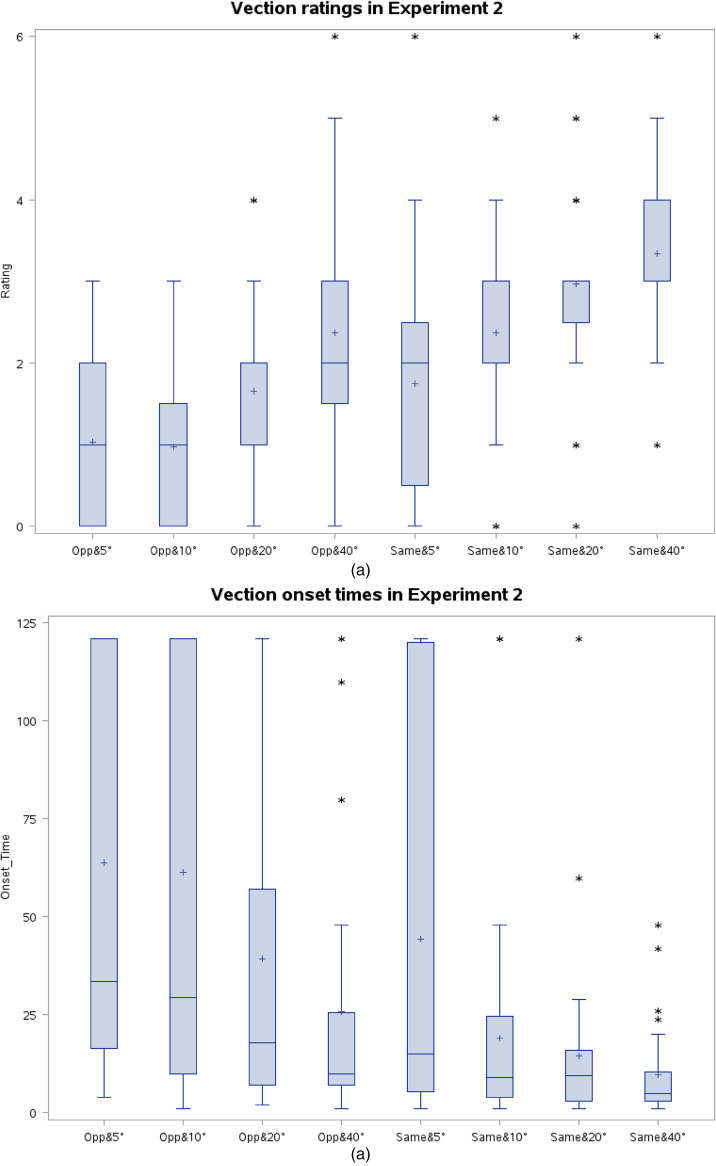
(a) Vection ratings for different stimuli directions and vertical stimuli FOVs in Exp2. Lower and upper edges of the box correspond to the lower and upper quartiles, respectively. The horizontal line in/on the box and cross represent the locations of the median and mean values respectively. The whiskers extend till the data point within ±1.5 interquartile range. * indicates one or more outliers of the same value. (b) Vection onset times for different stimuli directions and vertical stimuli FOVs in Exp2. Lower and upper edges of the box correspond to the lower and upper quartiles, respectively. The horizontal line in/on the box and cross represent the median and mean values, respectively. The whiskers extend till the data point within ±1.5 interquartile range. * indicates one or more outliers of the same value.

The dimensions of the vertical FOV affected vection onset times (*p* < .05; Friedman Test), and vection ratings (*p* < .05; Friedman Test). As shown in Figures 5(a) and (b), smaller vertical FOVs caused longer vection onset times and lower vection ratings in general.

For the conditions of 40° vertical FOV, almost all (i.e., 97%) of the trials resulted in vection. This percentage decreased with decreasing stimuli FOVs. For the stimuli that were going in the same direction and covering 5° vertical FOV, 75% of the trials ended in vection, and 88% of the subjects reported vection at least once with this minimal stimulation FOV. The total visual area of this condition is less than a quarter of the total visual area of two Apple Watches viewed at a distance of 40 cm. We should point out that the vection ratings for the 5° FOV conditions (median = 2) were lower than that of the stimuli FOV of 40°. A rating of 2 was defined as “I feel that I am moving but dots are moving more.” According to the subjects’ verbal descriptions, this feeling was the same as a real feeling of self-motion while observing a moving array of dots.

### Results of Experiment 3

Median values of (vection ratings, onset times) were (1, 16), (1, 11.5), (2, 14.5) for 30°, 45°, and 70° horizontal locations, respectively. Horizontal locations of 30°, 45°, and 70° did not result in significantly different vection onset times (*p* > .1; Friedman Test) (*p* > .1, d.f. = 2; ANOVA), and vection ratings (*p* > .1; Friedman Test) (*p* > .1, d.f. = 2; ANOVA). In other words, vection ratings and onset times for a pair of stimuli when placed at 30° horizontal locations on either side of the subject were similar to those when placed at 45° or 70°.

## Discussion

Held et al. (1975) found that increased speed causes higher levels of perception of tilt among observers of a rotating display. Andersen and Braunstein (1985) wrote that the reported self-motion duration reduced when the speed increased. In our Experiment 1, higher stimuli speed caused shorter vection onset times and higher vection ratings which are consistent with the literature. In Johansson's (1977) study, only 6.7% of the participants—1 out of 15—experienced vection while viewing the two stripes covering 47 square-degrees without frontal occlusion. In our Experiment 1, 94.2% of such trials resulted in vection. The probable reason for the stark difference between these percentages is the absence of ceiling light in our current study. In our earlier study (Dizmen & So, 2015) we found that ceiling light-off conditions caused higher vection ratings and shorter onset times compared to the ceiling light-on conditions. The high level of vection reporting and the consistency between our results and past literature suggests confidence in our experimental settings and results.

In Experiment 2, the moving dots pattern stimuli covering two narrow (1° width) and short (5° height) patches made 88% of the subjects experience vection. To the best of our knowledge, this is the first report of vection perception with visual motion within such a small FOV (i.e., 5 square-degrees for each eye). Therefore, from the scientific point of view, it is a very important finding that stimulation of such a small area on the retina results in vection. From the application point of view, on the one hand, the findings suggest caution in presenting visual motion in public areas (such as those on advertising panels) for fear that they may cause unwanted vection to observers and might affect their balance. On the other hand, the “grouping” effects may be useful for game developers to design self-motion cues in games. Subjects reported that they perceived the stimuli areas as small windows showing views of the outside-environment. This serves as an explanation to why a FOV of 5 square-degrees at each side of the observer can provoke sensations of vection.

In Exp1, the occlusion of the frontal vision with cardboard caused higher vection ratings and shorter vection onset times compared to staring at an LED in the dark room which had many static visual cues such as furniture. This may seem to be in conflict with the theory that the more static clues in the foreground, the stronger is the vection caused by background moving stimuli (Howard & Howard, 1994; Ohmi et al., 1987; Nakamura, 2006; Nakamura & Shimojo, 1999). But considering how small our stimuli were, the observers might have regarded the stimuli as part of the background when the frontal vision was occluded with the cardboard. The blocking of the central vision may provide the freedom for an observer to imagine that the environment behind the cardboard is moving too. The hypothetical explanation that subjects viewed the stimuli as part of a larger background moving scene was supported when subjects reported circular vection when they saw dots moving up on their left and down on their right (or vice versa). The subjects reported that they connected the two stimuli mentally and perceived them as parts of a single moving background. This is consistent with Palmisano and Gillam (1998)'s finding that when two peripherally-located motion stimuli are seen as being part of the same background scene, compelling vection is induced. But when these two motion stimuli are misperceived to be rotating about separate axes (as opposed to the same axis), then vection is destroyed. Our findings also support the perceptual grouping theory (Wagemans et al., 2012). First of all, the two stimuli followed the rule of similarity as they were of the same shape, size, and color. Secondly, the experimental setting provided opportunity for the rule of common fate to come into play, as the optic flows on both sides were moving in the same direction with the same speed in a synchronized way. Therefore, the participants perceived them as parts of a single entity.

By mentally connecting two stimuli, we do not only mean interpolation of the two stimuli in the shortest way in the front, but also extrapolation of them to the entire hidden visual field of the background to reach a visual closure of the entire background. For example, when the two stimuli are placed at 30° horizontal location, they span approximately 60° horizontal FOV in front of the subject; whereas 70° horizontal location spans approximately 140° horizontal FOV. We already proved that larger FOV causes more compelling vection (in Exp2). Therefore, if there were only interpolation of the two stimuli, 70° horizontal location would cause more compelling vection than 30° horizontal location because of the area spanned after interpolation. However, as the results of Exp3 showed, there is no significant difference in vection measures of 30° and 70° horizontal locations. Interpolation alone is not enough to explain this effect. Therefore, we have to accept that the subjects not only interpolate but also extrapolate the stimuli to a larger moving outer environment. In this way, we can explain why vection measures at 30° horizontal location are not different from those at 70° horizontal location: they do this because the two measures carry the same amount of information about the outer environment as long as their stimulation FOVs are also the same. Interpolation and extrapolation may only be applicable to stimuli of the same size. If the stimuli sizes differ, then the strengths of vection also differ. As shown in Exp1 and Exp2, the larger the stimuli area, the higher the vection ratings and the shorter the vection onset times.

### Conclusion and limitations

Johansson (1977) found that people who perceived vection while viewing a scene of moving dots through large FOVs continued to perceive vection after reducing the stimulation FOV to 47 square-degrees for each eye. Our study demonstrated that conditioning the observer with large stimulation FOVs is not necessary to induce vection with small stimulation FOVs. Two small stripes (1° horizontal by 5° vertical) of moving dots could cause an illusion of self-motion among 88% of the subjects of our Experiment 2. To our knowledge, this is the first report of vection induced by visual motion occupying such a small FOV in the eyes (Johansson, 1977; Kenyon & Kneller, 1993; Warren & Kurtz, 1992).

Results of our experiments also indicated that: (i) reducing the visual cues available by placing a cardboard in front of the observer caused higher levels of vection; (ii) faster stimuli (≈25°/sec) caused higher levels of vection compared to slower stimuli (≈5°/sec); and (iii) a pair of stimuli moving in the same direction caused linear vection whereas a pair of stimuli moving in opposite directions caused circular vection.

Observers perceptually integrated the two stimuli to perceive linear or circular vection depending on the stimuli directions.

The findings of this study are relevant to the design of mobile devices and wearable technology that usually have smaller display areas. The interfaces with large FOVs such as 3D movie theaters, motion simulators, or video games may also utilize our findings as synchronized motion of two stripes at the peripheral vision may cause vection and increase observers’ engagement levels. Designers must also keep in mind that prolonged vection may cause visually-induced motion sickness; and avoid endless streams of moving segments in the display areas that may be perceived as background motion. We hope that our findings will help in the process of designing more engaging user interfaces and displays with increased visual comfort.

In this study, we purposely avoided using large FOV vection stimuli so as not to cause transfer of learning. About 2/3 of our participants also took part in other vection studies using typical large FOV vection stimuli (Wei et al., 2018, 2019). Notwithstanding that, for future studies to further determine the minimum conditions for vection induction, it would be desirable to add a control condition with large FOV vection stimuli at the end of the experiment.
